# The Role of Processed Electroencephalography in the Detection and Management of Acute Cerebral Ischemia: A Scoping Review

**DOI:** 10.1097/ANA.0000000000001018

**Published:** 2025-01-09

**Authors:** David W. Hewson, Alex Mankoo, Philip M. Bath, Mark Barley, Permesh Dhillon, Luqman Malik, Kailash Krishnan

**Affiliations:** * Anaesthesia and Critical Care Medicine; ‡ Stroke Medicine; ¶ Interventional Neuroradiology, Queens Medical Centre, Nottingham University Hospitals NHS Trust; † University Department of Anaesthesia and Critical Care, Injury, Recovery and Inflammation Sciences, School of Medicine, University of Nottingham; § Stroke Trials Unit, School of Medicine, University of Nottingham, Nottingham, UK; ∥ Interventional Neuroradiology, Gold Coast University Hospital, Southport, QLD, Australia

**Keywords:** anesthesia, general [D000768], electroencephalography [D004569], ischemic stroke [D000083242], perioperative care [D019990], thrombectomy [D017131]

## Abstract

Processed electroencephalography (pEEG) is increasingly used to titrate the depth of anesthesia. Whether such intra-procedural pEEG monitoring can offer additional information on cerebral perfusion or acute focal or global cerebral ischemia is unknown. This scoping review aimed to provide a narrative analysis of the current literature reporting the potential role of pEEG in adults with acute cerebral ischemia. In keeping with the scoping review methodology, a broad search strategy was defined, including descriptions of encephalography in acute ischemic stroke, carotid endarterectomy, cardiac surgery, and cardiac arrest. Additional screening of citations was conducted by 2 independent assessors. From 310 records, 28 full-text articles met inclusion criteria. Most identified studies were observational in design, and described the diagnostic ability of pEEG to identify cerebral hypoperfusion or its prognostic sensitivity after stroke or carotid surgery. No studies were identified that evaluated pEEG in the specific setting of endovascular therapy for acute ischemic stroke. Low sensitivity associations between pEEG indices and cerebral blood flow were highlighted, which may be influenced by cerebral autoregulatory thresholds. Despite the associations reported in observational studies, this review identified significant uncertainty in the role of pEEG during cerebral ischemia. There is a paucity of high-level observational (cohort or case-control) or randomized trial research examining the possible role of pEEG for the detection and management of cerebral ischemia during acute stroke, including during endovascular therapy, or in other common scenarios of acute cerebral ischemia.

New acute focal or global cerebral ischemia may present before or during episodes of general anesthesia. An increasing number of patients with acute ischemic stroke (AIS) are offered endovascular therapy^1,2^ to achieve vessel recanalization, and therefore present for anesthetic care with rapidly evolving states of cerebral ischemia. In addition, patients undergoing carotid or cardiac surgery may experience rapid alterations of flow in the cerebral circulation, resulting in acute intraoperative cerebral ischemia. Finally, cerebral ischemia may result from globally reduced flow states, such as intraoperative hypotension, anaphylaxis, or cardiac arrest.

Processed electroencephalography (pEEG) provides a real-time graphical summation of the spontaneous local field potentials generated by cortical pyramidal neurons and detected by scalp electrodes^3^ typically placed on the forehead and recording signals from the prefrontal cortex. Characteristic patterns of pEEG oscillation are observed during general anesthesia.^4^ Intraoperative pEEG monitoring is advocated to monitor the depth of anesthesia during both cardiac and non-cardiac surgery, particularly during total intravenous anesthesia with neuromuscular blockade. pEEG guides anesthetic drug dosing and provides a direct measure of cerebral activity to indicate likely wakefulness, sedation, or unresponsiveness. An additional justification for perioperative pEEG monitoring is to provide indirect evidence of cerebral perfusion.^3^ The electroencephalogram (EEG) is highly sensitive to hypoxia, with frequency changes typified by asymmetrical decreased alpha and increased delta and theta activity in response to moderate or severe hypoxia.^5^ This allows for EEG interpretation of brain function and cerebrovascular metabolism.^6^ Although early assessments of the utility of near-infrared spectroscopy as a surrogate monitor of cerebral perfusion in patients with AIS have been reported,^7^ the role of pEEG in the diagnosis and management of AIS-related cerebral ischemia remains unclear.

The aim of this scoping review was to identify the extent and quality of the literature relating to pEEG monitoring in the diagnosis and management of acute cerebral ischemia, and thereby establish boundaries of knowledge and gaps in the current understanding of pEEG monitoring, including during the hyperacute phase of AIS patients.

## METHODS

This scoping review was performed according to the principles described by Levac and colleagues^8^ and conforms to the Preferred Reporting Systems for Systematic Reviews and Meta-Analyses Extension for Scoping Reviews (PRISMA-ScR) framework.^9^ As PROSPERO disallows the submission of scoping review protocols, this review was not pre-registered.

## DEFINITIONS

### Full Diagnostic Electroencephalography

A full EGG is usually performed with at least 16 channels to localize abnormalities in specific brain regions. Such testing is usually supervised and interpreted by clinical neurophysiologists and neurologists and may include specialist sleep, ambulatory, and video-telemetry modalities, most commonly for the investigation of epilepsy. The logistical impediments to full diagnostic EEG make it unlikely to be practical as a monitoring tool during acute episodes of cerebral ischemia, such as during endovascular therapy for AIS.

### Quantitative Electroencephalography

Quantitative EEG (qEEG) involves the processing, transformation, and analysis of EEG signals to present information on specific frequency bands, frequency distributions, neural connectivity, and networks. The resulting data are commonly displayed in graphical or diagrammatic format to facilitate interpretation. qEEG is used as a clinical and research tool in the evaluation of brain injury, epilepsy, and other neurological disorders. Its use in the assessment and prognostication of patients with AIS has been the subject of a recent comprehensive review.^10,11^

### Processed Electroencephalography

pEEG techniques use computerized EEG systems incorporating signal filtering, artifact rejection, and processing of frontal cortical EEG from 1 to 4 channels to derive proprietary numerical indices, which are in widespread use to aid clinical interpretation of frontal cortical electrical activity in the perioperative period. Several such systems are commercially available including Bispectral Index (BIS, Medtronic), Entropy (GE Healthcare), Sedline (Masimo Corporation), and Narcotrend (Narcotrend Gruppe). Each system has unique features, but broadly they apply proprietary algorithms, often incorporating Fourier waveform analysis, to record patterns of electrical activity from the frontal cortex. Systems typically display an EEG bio-signal, a proprietary dimensionless index calibrated around a probability of recall, measures of burst suppression, and electromyographic detection.

## OBJECTIVES

The 4 objectives of this scoping review are to:

1. Identify the characteristics (number and design) of studies examining the role of pEEG, full diagnostic EEG or qEEG systems in the management of patients sustaining cerebral ischemia from a range of aetiologies, including during hyperacute endovascular therapy for AIS, cardiac arrest, or endarterectomy (CEA).
2. Identify the configuration (manufacturer and indices) and usability of pEEG systems for the detection of acute cerebral ischemia.
3. Categorize studies by the intended purpose of pEEG monitoring in diagnosis (assessing acute neurological status or assisting in clinical decision-making), determining prognosis, or any other roles.
4. Perform a narrative analysis of the literature to identify knowledge gaps and offer recommendations for future research relating to pEEG monitoring during endovascular therapy for AIS.

## LITERATURE SEARCH

A structured search of the MEDLINE and EMBASE databases from inception to December 31, 2023 was conducted using the Ovid interface. The search keywords ‘stroke’, ‘carotid’, ‘ischemia’, ‘thrombectomy’, ‘endovascular therapy’, ‘electroencephalogram’, ‘bispectral, ‘narcotrend’, ‘sedline’, ‘entropy’ were applied with search truncation, wildcards, and Boolean operators. The strategy terms executed in the databases are shown in Supplemental Digital Content 1, http://links.lww.com/JNA/A785. To maximize understanding of the literature, tangential searching of the reference lists of identified studies was conducted, seeking studies encompassing common proprietary pEEG systems and also examinations of acute cerebral ischemia using full diagnostic EEG and/or qEEG. A supplemental search of the gray literature was conducted using Google Scholar. To minimize bias in the return of Google Scholar search results, the search was conducted using a web browser in private browsing mode. The first 100 Google Scholar records were reviewed for inclusion. Studies were limited to those reported in English and with full texts available. All clinical studies that addressed the use of pEEG in cerebral ischemia were included according to pre-defined population inclusion criteria: age older than or equal to 18 years, presenting with new acute cerebral ischemia, within 24 hours of ictus, posterior or anterior circulation stroke, and presuming any stroke severity. Studies pertaining to pEEG use during CEA or cardiac surgery were included as such procedures may provide a model for the cerebral response to AIS-related ischemia. In addition to clinical studies, review articles, opinion papers, case reports, and editorials were also included. Animal studies were excluded.

## SCREENING AND DATA EXTRACTION

All titles and abstracts identified by the initial search were de-duplicated and screened by 2 authors (A.M., D.W.H.) for detailed review according to the selection criteria. Discrepancies or uncertainties in data screening and extraction were resolved by consensus. Data on the following characteristics were extracted from included studies: authors, year of publication, title, country, primary objective, methodology, population, and nature of the pEEG device applied.

## RESULTS

**FIGURE 1:**
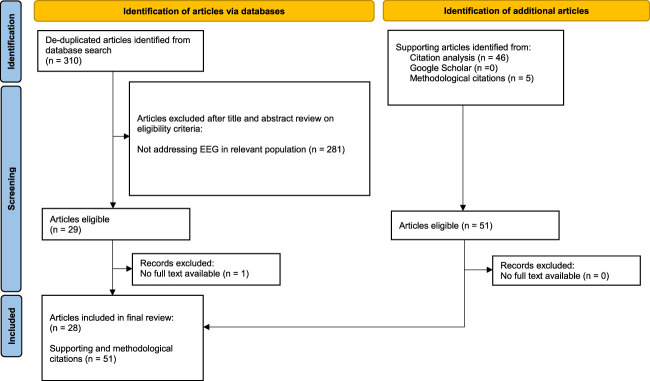
Flow diagram of literature search results and study selection.

A total of 310 nonduplicate records identified by the search underwent title and abstract screening of which 28 full-text articles^10,12–38^ were identified for inclusion in this review (Fig. 1). Manual searches of citations from identified studies by 2 authors (A.M., D.W.H.) returned an additional 46 articles providing supporting context to our scoping results. Together with methodological citations this yields a total of 79 articles included in this review.

The characteristics of the 28 full-text articles^10,12–38^ included in this review are summarized in Table 1. The literature is dominated by case reports,^12–23^ and observational studies.^24–35^ No prospective randomized trials assessing the utility of pEEG in patients with AIS were identified. Although the population of greatest interest for this scoping review was AIS, only 2 relevant articles describing the use of pEEG in AIS were identified, neither of which were performed on patients undergoing endovascular therapy.^33,34^ The remaining articles addressed the role of EEG in the relevant comparable populations of carotid endarterectomy,^12,13,16,20,24–32,36^ coil embolization of intracranial aneurysms,^17–19^ cardiac surgery,^14,15,21,35,37^ and non-cardiac surgery.^22,23,37,38^ To develop a more complete understanding of the state of evidence for this scoping exercise we included 8 studies addressing full diagnostic EEG,^10,12,28–31,36^ quantitative EEG^10^ alongside the modality of primary interest, and pEEG.^13–22,24–27,32–35,38^ In studies describing the use of pEEG in cerebral ischemia or stroke, we identified articles describing a variety of current and defunct proprietary systems, including BIS,^13–17,19,20,23–25,32–35^ Narcotrend,^21^ Entropy,^18,22^ Cerebral State Monitor,^26^ and Lifescan.^27^ In 5 studies, the pEEG system was applied for bi-hemispheric monitoring during non-cardiac,^22,23^ cardiac,^35^ and carotid surgery;^13,20^ in the remaining reports the pEEG systems were uni-hemispheric.

**TABLE 1:** Characteristics of Included Full-text Articles Reporting Use of Electroencephalography in Stroke, Cerebral Ischemia, or Carotid Surgery

First author, year, country	Design	Population	pEEG device	Main findings of relevance to pEEG in AIS
Harris et al, 1967, USA^12^	Case report	Carotid endarterectomy	NA—diagnostic EEG	First identified description of application of EEG during carotid endarterectomy for detection of hemispheric cerebral ischemic.
Sundt et al, 1981, USA^28^	Case series	Carotid endarterectomy	NA—diagnostic EEG	Use of EEG in 1145 carotid endarterectomies. Authors narratively report EEG changes related to the severity of reduction of cerebral blood flow determined by Xe^133^ tomographic technique.
McFarland, 1988, USA^29^	Case series	Carotid endarterectomy	NA—diagnostic EEG	Case series of 377 patients reporting use of EEG for identification of ipsilateral cerebral ischemia and determination of need for carotid shunt placement.
Silbert et al, 1989, Australia^27^	Case series	Carotid endarterectomy	Lifescan (Neurometics)	First identified description of application of processed EEG during carotid endarterectomy for detection of hemispheric cerebral ischemic.
Tempelhoff et al, 1989, USA^30^	Case series	Carotid endarterectomy	NA—diagnostic EEG with compressed spectral array	In 103 surgeries monitoring of EEG and spectral array was a reliable indicator need for carotid shunting by amplitude attenuation of the raw EEG and/or loss of high-frequency activity on spectral array.
Kearse et al, 1993, USA^31^	Case series	Carotid endarterectomy	NA—Diagnostic EEG with or without density spectral array	Description in 103 carotid endarterectomies of 16-channel analog EEG and density spectral array to detect cerebral ischemia.
Merat et al, 2001, France^38^	Case reports	Major vascular surgery	Bispectral index (A-2000 model)	Two case descriptions of decreases in BIS index observed during carotid endarterectomy and open repair of aortic aneurysm.
Welsby et al, 2003, USA^14^	Case report	Cardiac surgery	Bispectral index (A-2000 model)	Case description of perioperative embolic ischemic stroke arising during cardiac surgery identified by decrease in BIS index value.
Florence, 2004, France^37^	Narrative review	Adult non-cardiac and cardiac surgery	NA—diagnostic EEG	CBF decreases result in slowing and/or attenuation of EEG and processed EEG analysis is more sensitive than visual EEG analysis to detect cerebral ischemia. Decrease in α band or α and β bands power, together with increase in δ power is suggestive of brain hypoperfusion.
Deogonkar et al, 2005, UK^24^	Case series	Carotid endarterectomy	Bispectral index (A-2000 model)	In 52 patients there was lack of correlation between ipsilateral BIS index values and signs of cerebral ischemia. Authors conclude BIS index unreliable for detection of cerebrovascular insufficiency.
Bonhomme et al, 2007, Belgium^32^	Observational cohort	Carotid endarterectomy	Bispectral index (A-2000 model) and 5-lead EEG	In a total of 36 patients BIS index values were described to increase, stay constant or decrease in patients undergoing carotid cross-clamping. The authors caution the use of BIS index as a diagnostic tool for cerebral ischemia during carotid surgery.
Neema, 2009, India^16^	Case report	Carotid endarterectomy	Bispectral index (model not specified)	Description of increase in BIS index value in response to carotid shunt insertion and restoration of cerebral blood flow.
Kodaka et al, 2009, Japan^13^	Case report	Carotid endarterectomy	Bispectral index (A-2000, X-P model) applied in bi-hemispheric configuration	Description of increase in ipsilateral BIS index value in response to carotid shunt insertion and restoration of cerebral blood flow.
Sriganesh, 2009, India^17^	Case report	Intracranial vascular embolisation	Bispectral index (model not specified)	Description of decrease in BIS index value in response to cerebral vasospasm during arteriovenous malformation embolisation.
Lee et al, 2009, Korea^23^	Case reports	Non-cardiac surgery	Bispectral index (A-2000 model) applied in bi-hemispheric configuration	Description of interhemispheric BIS index value differences under general anesthesia in 2 patients undergoing non-cardiac surgery.
Kamath, 2009, India^18^	Case report	Intracranial vascular embolisation	State entropy	Description of profound decrease in entropy reading associated with carotid artery spasm during coil embolization.
Estruch-Perez et al, 2010, Spain^25^	Case series	Carotid endarterectomy	Bispectral index (A-2000 model)	In 80 patients undergoing endarterectomy a BIS index value decrease ≥14% from baseline had a sensitivity of 82% and specificity of 90% for cerebral ischemia.
Bleeker et al, 2010, Netherlands^15^	Case report	Cardiac surgery	Bispectral index (A-2000 model)	Case description of temporary decrease in BIS index value in response to brainstem transient ischemic attack.
Skordilis et al, 2011, USA^26^	Case series	Carotid endarterectomy	Cerebral state monitor	In 15 patients carotid clamping caused the ipsilateral CSI to drop below the control-side CSI. Shunt activation caused the CSI to rise above the control.
Khan, 2011, USA^22^	Case report	Non-cardiac surgery	State Entropy bi-hemispheric system	Description of interhemispheric differences in state entropy arising in patient with a history of carotid stenosis undergoing laparoscopic cholecystectomy.
Asaad, 2011, Egypt^35^	Observational cohort	Cardiac surgery in patients with or without carotid stenosis	Bispectral index bi-hemispheric configuration (VISTA model)	In patients with carotid stenosis undergoing cardiac surgery, aortic cross-clamping was associated with significant interhemispheric BIS index differences.
Rinoesl, 2013, Austria^21^	Case report	Cardiac surgery	Narcotrend	Case description of response in Narcotrend index values to cerebral ischemia during intraoperative aortic dissection.
Harclerode et al, 2013, UK^19^	Case report	Intracranial vascular embolization	Bispectral index (model not specified)	Description of repeated reversible decreases in BIS index value in response to iatrogenic balloon occlusion of the middle cerebral artery.
Zheng, 2018, Singapore^20^	Case report	Carotid endarterectomy	Bispectral index bi-hemispheric configuration (VISTA model)	Description of interhemispheric differences in BIS index in response to carotid cross-clamping.
Chang et al, 2020, USA^36^	Systematic review and meta-analysis	Carotid endarterectomy	NA—Diagnostic EEG	EEG changes have 46% sensitivity and 86% specificity for predicting perioperative stroke within 30 days of endarterectomy.
Akgol et al, 2020, Turkey^33^	Observational cohort	Acute stroke	Bispectral index (model not specified)	In 80 patients presenting to the emergency department with acute ischemic stroke, BIS index <74 at presentation was associated with subsequent in-hospital mortality with 94% sensitivity and 96% specificity.
Ozdemir, 2021, Turkey^34^	Observational cohort	Acute stroke	Bispectral index (model not specified)	In 43 patients with acute ischemic stroke presenting mean (standard deviation) BIS index value was 84 (9.5), but there was no significant correlation between BIS index and the NIHSS score.
Sutcliffe et al, 2022, UK^10^	Scoping review	Acute stroke	NA—full diagnostic and quantitative EEG systems described	Identified 39 articles evaluating diagnostic and/or prognostic implications of full diagnostic and quantitative EEG. No pEEG systems reported.

AIS indicates acute ischemic stroke; CBF, cerebral blood flow; CSI, cerebral state index; NA, not applicable; NIHSS, National Institutes of Health Stroke Scale; pEEG, processed electroencephalography.

## DETECTION OF PERIPROCEDURAL CEREBRAL ISCHEMIA

The use EEG monitoring for detection of intraoperative cerebral ischemia during CEA was first described in 1967,^12^ only 15 years after the first CEA was performed by Micheal DeBakey. Physiological data linking EEG changes to decreases in cerebral blood flow, and evidence that EEG data could influence clinical decisions on the placement of intraoperative shunts,^37^ meant that EEG monitoring was extensively examined in the literature over the subsequent 20 years. Nevertheless, the role of EEG monitoring alongside or in place of other indicators of neurological status (including clinical assessment in an awake patient, regional blood flow monitoring, stump pressure, transcranial Doppler monitoring, cerebral oximetry, and somatosensory evoked potentials) during surgery performed under general anesthesia remains a subject of controversy. Meta-analysis of data from 10,672 patients undergoing CEA enrolled in 35 trials indicated that EEG changes predict perioperative stroke with 46% sensitivity and 86% specificity.^36^ Given the low sensitivity and complexity of interpretation in a perioperative clinical environment, it is unsurprising that full diagnostic EEG monitoring is not widely advocated for the detection of cerebral ischemia during CEA.

The implementation of pEEG systems in modern anesthetic care, and their reported ability to identify cerebral ischemia during CEA,^26,27,30,38–41^ intracranial vascular procedures,^19^ before^15^ and during cardiopulmonary bypass,^14,21,35,42^ and after cardiac arrest^43^ suggests that iatrogenic cerebral ischemia induced by carotid cross-clamping during CEA represents a useful model of ischemic stroke to assess the diagnostic accuracy and reliability of pEEG. In 52 patients undergoing awake CEA, Deogaonkar and colleagues^24^ found no meaningful correlation between BIS index values and clinical evidence of cerebral ischemia during carotid cross-clamping and concluded that BIS monitoring during awake CEA is unreliable for the detection of ischemia in comparison to intraoperative neurological testing. It was subsequently observed in 26 patients undergoing CEA with general anesthesia that ipsilateral BIS index values may decrease, increase, or not change in response to carotid cross-clamping.^32^ Using transcranial Doppler ultrasonography as the comparator standard, Dahaba and colleagues^44^ assessed the discriminative power of bi-hemispheric BIS to detect reductions in middle cerebral artery blood flow velocity during ipsilateral carotid cross-clamping. Although BIS showed good correlation and discriminative power to detect significant reductions in middle cerebral artery blood flow velocity, the changes in BIS value were observed globally, with no significant interhemispheric difference. The authors concluded that, in contrast to transcranial Doppler, bi-hemispheric BIS is not a reliable indicator of cerebral ischemia. This conclusion accords with a prospective study of 80 patients undergoing CEA with local anesthesia which reported a relative BIS index decrement of 14% to have a 96.8% (95% CI, 88%-99.4%) negative predictive value for the detection of cerebral ischemia, but a positive predictive value of only 56.3% (95% CI, 30.6%-79.2%).^25^ In other words, a stable BIS index value suggests that ischemia is unlikely, but a fall in BIS value does not always indicate cerebral ischemia. Seemingly paradoxical increases in BIS index value have been reported in response to carotid clamping, shunting, and reperfusion,^20^ leading to speculation that neuronal excitability arising from borderline ischemia, decreased hemispheric anesthetic drug concentration or nociceptive stimulation from cross-clamping might be responsible.

Below the lower limit of autoregulation, cerebral perfusion is fundamentally affected by systemic mean arterial blood pressure (MAP).^45^ It is possible that the relationship between pEEG-derived values and cerebral perfusion only becomes manifest at or below the autoregulatory blood pressure threshold. To test this assumption, Liu and colleagues^46^ measured BIS index values and estimated the lower limit of cerebral autoregulation by determining the MAP and cerebral blood flow velocity in 79 patients undergoing stable general anesthesia for cardiac surgery. They observed a dose-response effect, with BIS values decreasing in parallel with increased burst suppression and in proportion to the MAP decrement below the lower limit of autoregulation; however, BIS values were unchanged when MAP was maintained above autoregulation thresholds. By sampling data during cardiopulmonary bypass with constant hypnotic drug dosing, the authors were able to reduce confounding from the effect of varying does of anesthetic agents. This finding agrees with published cases describing a similar phenomenon,^47^ and an animal model of hemorrhagic shock which demonstrated changes in BIS value in response to cerebral hypoperfusion independent of plasma propofol concentrations.^48^

In summary, sudden reductions in pEEG indices during iatrogenic models of reduced cerebral blood flow, specifically the BIS index value, are weakly associated with cerebral ischemia. However, the positive predictive value of such changes is probably low. This is unsurprising given the known (and indeed intended) influence of hypnotic medication on pEEG index values. When controlling for varying doses of hypnotic medication, pEEG index values seem to fall in response to altered cerebral metabolic activity arising from global cerebral ischemia when cerebral blood flow is below autoregulatory thresholds. Although BIS was the most commonly reported pEEG system identified in our literature search, similar reports using the Entropy^18,22^ and Narcotrend^21^ systems were identified. The more rapid response time of Narcotrend values to alterations in depth of anesthesia^49^ may mean that Narcotrend is a more useful system to rapidly identify changes in cerebral blood flow and ischemia.

## PROGNOSTICATION AFTER STROKE

Disordered consciousness, arising from direct or indirect injury to the arousal centers of the brainstem and thalamus, is common after AIS and predicts morbidity and mortality.^50^ A large variety of clinical assessments,^51^ biomarkers,^52^ and electrophysiological,^53^ and neuroimaging^54^ techniques have been proposed to evaluate consciousness and predict the likelihood of recovery of wakefulness and/or longer-term outcomes. Although BIS index values measured on hospital admission were not correlate directly with Glasgow Coma Score in patients presenting with altered consciousness arising from traumatic or non-traumatic causes,^55^ BIS index has been shown to predict outcomes after cardiac arrest,^56^ and to differentiate patients who recover consciousness after severe brain injury from those who do not.^57^

pEEG has therefore been the subject of investigation in relation to prognostication after stroke. In 102 subjects with post-AIS disordered consciousness, a 4-item composite scoring system of age, BIS index, motor response and brainstem response assessed on day 3 after ictus produced good predictive ability to identify patients who will recover consciousness, with an area under the curve of 0.931 (95% CI, 0.882-0.980).^58^ In predicting in-hospital mortality after AIS, BIS index value at time of hospital presentation has an area under the curve of 0.984 (95% CI, 0.926-0.999).^33^ A cutoff BIS index value of ≤ 74 predicted in-hospital mortality after stroke with 93.6% (95% CI, 78.6%-99.2%) sensitivity and 95.9% (95% CI, 86.0%-99.5%) specificity, prognostic performance broadly equivalent to Glasgow Coma Score assessment. Such prognostication is described in non-anesthetised subjects who are not concurrently undergoing re-vascularization therapies, so translation into a hyperacute endovascular therapy setting is uncertain.

Although the National Institutes of Health Stroke Scale (NIHSS) is primarily used to quantify post-stroke impairment and assess treatment efficacy, the scale is also strongly predictive of 3-month outcome in some, but not all, stroke subpopulations.^59^ Unsurprisingly, given that only one scoring domain of the NIHSS relates directly to consciousness, there is limited evidence that pEEG index values correspond to the NIHSS. Whereas Özdemir and colleagues^34^ reported no relationship between NIHSS and BIS index values in 43 patients after AIS, another study in 53 patients with anterior circulation stroke found that NIHSS at hospital discharge was inversely correlated with BIS index values measured after intravenous reperfusion therapy within 6 hours of hospital admission.^60^

## DETECTION OF STROKE-RELATED COMPLICATIONS

Continuous EEG and/or qEEG are essential diagnostic tools to detect seizures and delineate duration and response to therapy in critically ill patients. Continuous EEG is also advocated internationally for the identification of delayed cerebral ischemia in comatose patients after subarachnoid hemorrhage.^61^ Eight-channel diagnostic EEG is currently subject to investigation as a pre-hospital tool to detect large vessel occlusion strokes suitable for direct transfer to neuroscience centers for endovascular therapy.^62^ In contrast to those modalities, pEEG monitoring is not currently recommended for such indications. Several studies have reported use of pEEG to detect seizures during general anesthesia, but these have conflicting results. Some studies reported an increase in BIS index,^63^ whereas other others reported either no change^64^ or a decrease in BIS.^65^ It has been suggested that pEEG indices interpret the high-frequency disordered electrical activity of seizures as artifact.^66^ In one study applying pEEG in patients with known epilepsy in an ambulatory setting, readily noticeable changes in the BIS-derived density spectral array and electromyogram occurred during both non-convulsive and convulsive seizures during wakeful or natural sleep epochs. However, the authors of that study reported no significant changes in the BIS index.

## ACCEPTABILITY OF PEEG MEASUREMENT DURING ENDOVASCULAR THERAPY

The acceptance of pEEG monitoring during endovascular therapy requires a clear clinical and/or cost benefit to be balanced against the potential harms associated with its use. This scoping review has indicated that the utility of pEEG monitor in the setting of AIS and during reperfusion therapy remains a subject requiring systematic investigation. Conversely, the theoretical harms of pEEG monitoring can be elucidated based on the clinical experience of its use in the wider perioperative setting. Skin electrode pEEG devices are non-invasive and painless to apply and maintain. Standard frontotemporal electrode positions are only inaccessible for monitoring in circumstances of pre-existing injury or planned surgery to that area. In adult patients, adequate electrode spacing and impedance can be accomplished in virtually all cases. Placement of electrodes and their connecting lead, the calibration of the pEEG device (e.g., provision of demographic information for the Narcotrend), and confirmation that signaling has successfully commenced require the attention of the supervising clinician and some time, likely 1 or 2 minutes, to execute. Given that endovascular therapy for AIS is provided on a time-critical basis (‘time-is-brain’), clinicians must be cognizant of this imposition and be certain that pEEG monitoring will not result in a meaningful delay in obtaining vessel recanalization in an individual case. Our literature search failed to identify data quantifying these processes or assessing the acceptability of pEEG monitoring among users. As with the general perioperative setting, it is likely that pEEG signals obtained during endovascular therapy could be subject to electromyographic,^57,67^ electrocardiographic, and direct external electrical interference.^68^ Careful interpretation of such interference is needed to distinguish meaningful pEEG changes from those arising from signal noise.

## UNI-HEMISPHERIC VERSUS BI-HEMISPHERIC DATA ACQUISITION

pEEG monitors are available in configurations that provide either uni-hemispheric or bi-hemispheric signal acquisition. For the purposes of titration of anesthetic drugs to maintain a constant depth of anesthesia, uni-hemispheric signal acquisition is typically undertaken as concordance between the hemispheres typically exceeds 70%.^69^ However, the Sedline algorithm uses bi-hemispheric concordance, necessitating a 4-channel bifrontal electrode to identify an anesthetised hypnotic state. Bi-hemispheric monitoring may also provide information on differences in electrical activity between hemispheres of relevance in ischemic stroke and during endovascular therapy. Novel 2-channel EEG-derived markers of ischemic stroke such as the lateral interconnection ratio (a measure of interhemispheric symmetry) are being developed and tested for performance in patients with known stroke compared with non-stroke patients during general anesthesia.^70^ Bi-hemispheric monitoring has been evaluated in patients with known unilateral supratentorial pathology including subarachnoid hemorrhage, intracerebral hemorrhage, and ischemic stroke. In an observational study of twenty such patients undergoing sedation and mechanical ventilation, a lower mean (standard deviation) BIS index value was reported in the hemisphere of mixed intracranial pathology compared with the contralateral healthy hemisphere (34.7 [14.6] vs. 41.2 [15.2]).^71^ Although similar findings of interhemispheric differences in BIS index have been reported during critical illness in adults^72^ and in children,^73,74^ and during general anesthesia for neurological^75^ and non-neurological^23,69,76^ procedures, the clinical relevance of these interhemispheric difference is unknown and these results have not been universally replicated.^77^

## DISCUSSION

This review summarizes the current state of evidence in relation to the use of pEEG in the detection and/or management of acute cerebral ischemia. A summary of the scoping findings and suggestions for future research specifically for patients undergoing endovascular therapy after AIS is found in Table 2. We identified scientific literature reporting the utility of EEG systems for the detection of periprocedural cerebral ischemia, prognostication after stroke, the detection of ischemic complications such as seizures, and interhemispheric differences in pEEG data in the presence of cerebral pathology. Many of the identified studies were conducted on comparable populations relevant to stroke, including carotid stenosis and endarterectomy, traumatic brain injury, subarachnoid hemorrhage, and critical illness. The extent to which these findings translate to large vessel occlusion and AIS is uncertain.

**TABLE 2:** Scoping Review Summary and Knowledge Gaps/Future Research Questions

Summary of scoping review findings
Twenty-eight articles, almost all case series or small cohort designs, describe EEG monitoring in clinical scenarios of acute cerebral ischemia (e.g., during AIS or carotid endarterectomy).
The numerical response of proprietary pEEG indices to uni-hemispheric cerebral ischemia seems to be unpredictable, with a low positive predictive value.
As a constituent of composite scores, pEEG indices may assist in the prognostication of patients presenting with AIS.
No published studies describing the utility of pEEG during EVT for AIS were identified.
Systematic descriptions of pEEG signal changes (i.e., looking beyond index values) arising during AIS and by EVT were absent in the literature
Knowledge gaps and proposed future research questions for use of pEEG during EVT for AIS
What are the barriers and facilitators to implementation and testing of pEEG systems in the hyperacute setting, and do these render prospective research on this topic non-feasible?
Does bi-hemispheric frontal pEEG monitoring reliably distinguish ischemic and non-ischemic cerebral hemispheres in large vessel occlusion AIS?
Is there a role for pEEG in guiding clinical decisions on use of local anesthesia, conscious sedation, or general anesthesia during EVT?
Do pEEG signals change in response to large vessel recanalization and restoration of cerebral blood flow and oxygenation during EVT or carotid endarterectomy?
Can pEEG-derived data inform intraprocedural management of arterial blood pressure, or decisions on adjuvant perfusion therapies such as intra-arterial thrombolysis?

AIS indicates acute ischemic stroke; EVT, endovascular thrombectomy; pEEG, processed electroencephalography.

Of greatest relevance to endovascular therapy for AIS, there is no direct evidence regarding the reliability of pEEG to identify changes in hemispheric cerebral oxygenation in stroke patients undergoing re-vascularization. Whether emergency reperfusion during endovascular therapy produces pEEG signal change, and whether this information is of clinical utility to guide treatment or inform prognosis, is unknown. Given that approximately half of stroke patients experience poor functional outcome despite complete recanalization, it has been postulated that persistent areas of cerebral hypoperfusion after endovascular therapy—the so-called ‘no-reflow’ phenomenon—could explain this apparent therapy failure. No-reflow may occur due to micro-emboli formation and distal emboli shower occluding small vessels at the arteriole and capillary level. It is currently uncertain whether adjunctive intravenous or intra-arterial thrombolytic or antiplatelet therapy improves perfusion through the most distal vasculature and subsequently improves functional outcomes. The utility of future intraprocedural monitoring devices, including pEEG, may lie in the evaluation of tissue-level physiological responses to large vessel intracranial recanalization (alongside radiologic and/or clinical assessments) and to guide choices of adjuvant perfusion therapies. Furthermore, end-user acceptability of pEEG monitoring in hyperacute/emergency stroke pathways has not been formally reported. Acceptability could be assessed by the ability of clinicians to obtain pEEG signals in emergency endovascular therapy situations, the completeness of pEEG signal acquisition, and clinician-reported feedback on the extent to which pEEG signals impact on intraprocedural decision-making. The extent to which pEEG may guide clinicians in rapid decisions to either sedate or anesthetize specific patients during endovascular therapy, a topic of international variability and ongoing controversy,^2,78,79^ is also unknown. A limitation of pEEG monitoring is that the bio-signal is retrieved only from the prefrontal cortex. Intuitively one would expect altered cerebral electrical activity primarily in those territories subject to the ischemic event, which may limit pEEG devices to detection of signal change from anterior cerebral artery and superior division middle cerebral artery thromboembolic events.

The widespread perioperative experience of pEEG confirms that available commercial systems can be almost universally applied with almost no contraindications to their use. Furthermore, their indices (if not the unprocessed EEG waveforms concurrently displayed on devices) are easily interpreted, even if signal artifact from frontal muscle contamination and/or external electromagnetic interference is relatively common. On the basis of current published literature, additional research into the utility of pEEG in patients with AIS undergoing emergency re-vascularization is needed. The lengthening time window after ictus during which endovascular therapy is being conducted, and the expanding indications for intervention to include patients with large-core infarcts, mean that the volume of endovascular interventions is likely to rise substantially during the next decade. Whether pEEG can assist in clinical decision-making and prognostication has not been definitively answered and further investigation is urgently warranted.

## Supplementary Material

SUPPLEMENTARY MATERIAL
