# Supplementary material for: The Role of Processed Electroencephalography in the Detection and Management of Acute Cerebral Ischemia: A Scoping Review
Source: J Neurosurg Anesthesiol. 2025 Jan 9;38(1):43–52. doi: 10.1097/ANA.0000000000001018 (PMC12662142; doi:10.1097/ANA.0000000000001018)
Supplement: SUPPLEMENTARY MATERIAL [file ana-38-43-s001.pdf]

Supplemental digital content 1: Search strategy terms executed in databases

Embase <1974 to 2023 Dec 31>
Ovid MEDLINE(R) ALL <1946 to Dec 31, 2023>

1	stroke.m_titl.	345759
2	thrombectomy.m_titl.	19828
3	endovascular.m_titl.	77736
4	"electroencephalo*".m_titl.	32462
5	bispectral.m_titl.	3843
6	narcotrend.m_titl.	160
7	sedline.m_titl.	38
8	entropy.m_titl.	18806
9	carotid.m_titl.	140824
10	"isch*emia".m_titl.	199579
11	1 or 2 or 3 or 9 or 10	752311
12	4 or 5 or 6 or 7 or 8	54632
13	11 and 12	753
14	limit 13 to english language	650
15	limit 14 to human	490
16	limit 15 to humans	490
17	remove duplicates from 16	310
